# Blackcurrant Anthocyanins Increase the Levels of Collagen, Elastin, and Hyaluronic Acid in Human Skin Fibroblasts and Ovariectomized Rats

**DOI:** 10.3390/nu10040495

**Published:** 2018-04-16

**Authors:** Naoki Nanashima, Kayo Horie, Hayato Maeda, Toshiko Tomisawa, Maiko Kitajima, Toshiya Nakamura

**Affiliations:** 1 Department of Bioscience and Laboratory Medicine, Hirosaki University Graduate School of Health Sciences, 66-1 Hon-cho, Hirosaki, Aomori 036-8564, Japan; k-horie@hirosaki-u.ac.jp (K.H.); toshiyan@hirosaki-u.ac.jp (T.N.); 2 Faculty of Agriculture and Life Science, Hirosaki University, 3 Bunkyo-cho, Hirosaki, Aomori 036-8561, Japan; hayatosp@hirosaki-u.ac.jp; 3 Department of Nursing Sciences, Hirosaki University Graduate School of Health Sciences, 66-1 Hon-cho, Hirosaki, Aomori 036-8564, Japan; tmtott@hirosaki-u.ac.jp (T.T.); kitajima@hirosaki-u.ac.jp (M.K.)

**Keywords:** anthocyanin, blackcurrant, ovariectomized rat, phytoestrogen, skin

## Abstract

Blackcurrants (*Ribes nigrum* L.) contain high levels of anthocyanin polyphenols, which have beneficial effects on health, owing to their antioxidant and anticarcinogenic properties. Phytoestrogens are plant-derived substances with estrogenic activity, which could have beneficial effects on the skin. Estradiol secretion decreases during menopause, reducing extracellular matrix (ECM) component production by skin fibroblasts. Using a normal human female skin fibroblast cell line (TIG113) and ovariectomized rats, the present study investigated whether an anthocyanin-rich blackcurrant extract (BCE) and four blackcurrant anthocyanins have novel phytoestrogenic activities that could benefit the skin in menopausal women. In TIG113 cells, a microarray and the Ingenuity^®^ Pathway Analysis showed that 1.0 μg/mL of BCE upregulated the expression of many estrogen signaling-related genes. A quantitative RT-PCR analysis confirmed that BCE (1.0 or 10.0 μg/mL) and four types of anthocyanins (10 μM) altered the mRNA expression of ECM proteins and enzymes involved in ECM turnover. Immunofluorescence staining indicated that the anthocyanins stimulated the expression of ECM proteins, such as collagen (types I and III) and elastin. Dietary administration of 3% BCE to ovariectomized rats for 3 months increased skin levels of collagen, elastin, and hyaluronic acid. This is the first study to show that blackcurrant phytoestrogens have beneficial effects on skin experimental models.

## 1. Introduction

Blackcurrants (*Ribes nigrum* L., Grossulariaceae) contain high concentrations of flavonoids, a group of polyphenolic compounds that includes anthocyanins and flavonols. Blackcurrants are reported to contain four anthocyanins: cyanidin-3-glucoside (C3G), cyanidin-3-rutinoside (C3R), delphinidin-3-glucoside (D3G), and delphinidin-3-rutinoside (D3R) [1]. Blackcurrant anthocyanins have a number of health benefits, such as improving blood flow and preventing breast cancer [1,2]. However, there have been no reports on their health benefits for the skin.

The skin is affected by estradiol [3,4,5]. Collagen, a fibrous protein, is the major constituent of the dermis, and, combined with elastin, retains skin elasticity and flexibility; these features decline during menopause, when the dermis loosens and the skin’s surface wrinkles and sags [3,4,5,6]. Matrix metalloproteinases (MMPs) degrade extracellular matrix (ECM) proteins, such as collagen and elastin, and tissue inhibitors of MMPs (TIMPs) inhibit this activity [7]. These enzymes also modulate the extracellular milieu. In addition to collagen and elastin, hyaluronic acid is abundant in the dermis; it retains moisture and thus determines the moisture content of the skin [8]. Hyaluronic acid is synthesized by hyaluronic acid synthase (HAS) [9]. Estrogen is involved in the regulation of the metabolism of ECM components, and because the level of this hormone declines markedly during menopause, the levels of ECM components, such as collagen [10,11,12], hyaluronic acid [13,14,15], and elastin [3,16], have been reported to decrease. Therefore, skin aging is promoted in menopausal women, and hormone replacement therapy can improve skin thickness in these individuals [11,17].

Phytoestrogens are a chemically diverse group of plant compounds with estrogenic effects in animals. Phytoestrogens, which include isoflavones, lignans, coumestans, and resveratrol, are present in many foods [18,19,20,21]. Anthocyanins, such as cyanidin and delphinidin, may also have phytoestrogenic activity [22]. Furthermore, we previously reported that blackcurrant anthocyanins act as phytoestrogens both in vitro and in vivo [23,24]. Although phytoestrogens [14,25,26,27] and phytochemicals [28,29] have been reported to improve dermal health and increase the production of collagen, elastin, and hyaluronic acid, the effects of blackcurrant anthocyanins on these skin components have not been reported.

The objective of this study was to investigate the beneficial effects of an anthocyanin-rich blackcurrant extract (BCE) and four blackcurrant anthocyanins on the skin owing to their phytoestrogenic activity. We exposed fibroblast cells derived from normal human female skin (TIG113) to BCE or anthocyanins and assessed the changes in ECM gene and protein expression using microarrays, Ingenuity^®^ Pathway Analysis (IPA), quantitative RT-PCR (RT-qPCR), and immunofluorescence staining. Furthermore, we evaluated the effects of blackcurrant anthocyanin phytoestrogenic activity on the skin using ovariectomized (OVX) rats, which do not secrete estrogen.

## 2. Materials and Methods

### 2.1. Materials and Cell Culture

The BCE powder, CaNZac-35, was purchased from Koyo Mercantile Co. (Tokyo, Japan). Our previous study showed that BCE contains high concentrations of polyphenols (37.6 g/100 g BCE) and anthocyanins (38.0 g/100 g BCE): C3G (5.6%), C3R (32.0%), D3G (16.8%), and D3R (45.3%) [24]. C3G, C3R, D3G, and D3R were purchased from Nagara Science (Gifu, Japan). 17β-estradiol (E2) was purchased from Sigma-Aldrich (St. Louis, MO, USA). The TIG113 skin fibroblast cell line was obtained from the Health Science Research Resources Bank, Osaka, Japan. Cells were maintained in Dulbecco’s modified Eagle’s medium (DMEM) (Wako, Japan) with 10% fetal bovine serum, 100 units/mL penicillin, and 100 μg/mL streptomycin (Wako, Japan). All cell culture experiments were conducted at 37 °C in a humidified incubator containing 5% CO_2_.

### 2.2. Microarray Gene Expression Profiling

TIG113 cells were seeded in 21-cm^2^ culture dishes and grown under the conditions described in Section 2.1, until they reached confluence. The medium was then replaced with phenol red- and serum-free DMEM, with or without BCE (1.0 μg/mL). After a 24-h incubation period, the cells were washed twice with phosphate-buffered saline (PBS), and total RNA was extracted using the RNeasy mini kit (Qiagen, Hilden, Germany). RNA labeling and hybridization were performed using the Agilent One-Color Microarray-Based Gene Expression Analysis protocol (version 6.5, 2010; Agilent Technologies, Santa Clara, CA, USA). Briefly, 100 ng of total RNA from each sample was linearly amplified and labeled with Cy3-dCTP. The resultant labeled cRNAs were purified using the RNAeasy mini kit (Qiagen, Hilden, Germany). Labeled and fragmented cRNA was hybridized to a SurePrint G3 Human Gene Expression microarray (8 × 60 K version 3; Agilent Technologies, Santa Clara, CA, USA). Labeling, hybridization, image scanning, and data analysis were performed at Macrogen Japan Corp. (Tokyo, Japan). The microarray dataset is available at http://www.ncbi.nlm.nih.gov/geo, under the accession code, GSE102896.

### 2.3. IPA

Genes showing up or downregulation of ≥1.5-fold following the exposure of TIG113 cells to 1.0 μg/mL BCE were analyzed using IPA software (version 36601845). The z-score algorithm was utilized to reduce the possibility of false positive results, where z ≥2.0 indicated that transcript expression was significantly increased and z ≤−2.0 indicated that expression was significantly decreased.

### 2.4. RT-qPCR

TIG113 cells were seeded in 9-cm^2^ culture dishes and cultured as described in Section 2.1, until confluent. The medium was then replaced with phenol red- and serum-free DMEM, with or without anthocyanins (10 μM) or BCE (1.0 or 10.0 μg/mL). The cells were incubated for 24 h and then washed twice with PBS. Total RNA was extracted using the RNeasy mini kit (Qiagen, Hilden, Germany). cDNA was reverse-transcribed from total RNA (200 ng) using the PrimeScript™ RT Master Mix (TaKaRa, Tokyo, Japan). Levels of specific mRNAs were quantified by qPCR using GoTaq^®^ Green Master Mix (Promega Tokyo, Japan). PCR amplification consisted of 30 s at 94 °C, 30 s at 55–60 °C, and 30 s at 72 °C for 40 cycles. Transcript levels were normalized to those of glyceraldehyde 3-phosphate dehydrogenase (*GAPDH*). The primers were as follows (5′ → 3′): collagen type I alpha 1 chain (*COL1A1*), forward (F) TTGCTCCCCAGCTGTCTTAT and reverse (R) AGACCACGAGGACCAGAGG; collagen type III alpha 1 chain (*COL3A1*), F-TTGAAGGAGGATGTTCCCATCT and R-ACAGACACATATTTGGCATGGTT; elastin (*ELN*), F-GGCCATTCCTGGTGGAGTTCC and R-AACTGGCTTAAGAGGTTTGCCTCCA; hyaluronan synthase 3 (*HAS3)*, F-CTTAAGGGTTGCTTGCTTGC and R-CTTAAGGGTTGCTTGCTTGC; hyaluronoglucosaminidase 3 (*HYAL3*), F-TGCTGGCATCTCCATGACTACC and R-CTTCCATCTGTCCTGGATCTCG; *MMP12*, F-TGGTTTGGTTGTTAGAAATGGTGTA and R-CTGAGGACATAGCAAATATGCAATAAA; *TIMP3*, F-AGGCAAGCAGCTAGACTGGTGAA and R-AACTGGATGGGCAGCAGGAC; *GAPDH*, F-TGAGAACGGGAAGTCTGTCA and R-TCTCCATGGTGGTGAAGACG. PCR specificity was verified by melting curve analysis. All samples were analyzed in duplicate, and relative gene expression was calculated by the 2^−ΔΔ*C*t^ method.

### 2.5. Immunofluorescence Staining

Cells were seeded onto a 4-well Slide & Chamber (Watson, Japan) and incubated at 37 °C for 24 h before fixing immediately in 4% paraformaldehyde and permeabilizing with 0.1% Triton X-100 in PBS for 5 min. The slides were then incubated with the anti-COL1A1 (3G3) antibody (sc-293182, Santa Cruz Biotechnology, Santa Cruz, CA, USA, 1:50 dilution, *v*/*v*), anti-COL3A1 (B-10) antibody (sc-271249, Santa Cruz Biotechnology, Santa Cruz, CA, USA, 1:50 dilution, *v*/*v*), or anti-elastin (BA-4) antibody (sc-58756, Santa Cruz Biotechnology, Santa Cruz, CA, USA, 1:50 dilution, *v*/*v*) at 4 °C overnight, followed by incubation with the secondary antibody, Alexa Fluor^®^ 546 anti-mouse IgG (Life Technologies, Carlsbad, CA, USA, A11060, dilution 1:500) for 30 min at room temperature. Nuclear staining and mounting were performed using VECTASHIELD^®^ Mounting Medium with DAPI (Vector Laboratories, Burlingame, CA, USA). Images were captured using a FSX100 fluorescence microscope (Olympus, Tokyo, Japan).

### 2.6. Animals and Treatments

OVX female Sprague–Dawley and sham surgery rats, aged 12 weeks, were purchased (CLEA Japan, Inc., Tokyo, Japan) and group housed in plastic cages in air-conditioned rooms with a 12-h light/dark cycle at the Institute for Animal Experiments of Hirosaki University Graduate School of Medicine. The Animal Research Committee of Hirosaki University approved this study (permission number: G16004), which was conducted in accordance with the rules for animal experimentation of Hirosaki University. Our previous studies have shown that 3% BCE exerts phytoestrogenic effects in rats [24]. All of the rats received the AIN-93M diet, with and without 3% BCE, as indicated (Oriental Yeast Co., Ltd., Tokyo, Japan) and were divided into 3 groups: (1) OVX rats treated with 3% BCE for 3 months (*n* = 3), (2) OVX rats without BCE treatment (control) (*n* = 4), and (3) sham surgery rats without BCE treatment (*n* = 3). All rats had free access to water and food. At the end of the experiment, the animals were euthanized, and back skin was removed. Uterine tissue was fixed in 10% formaldehyde and embedded in paraffin for histological examination. Skin tissue sections (4-μm thick) were routinely passed through xylene and a graded alcohol series before Masson’s trichrome staining, elastic fiber staining, and immunohistochemical analysis of hyaluronic acid.

### 2.7. Collagen Thickness Measurement

To evaluate connective tissue thickness, sections were stained with Masson’s trichrome stain. The specimens were examined and photographed using a digital microscope camera (DP21, Olympus, Tokyo, Japan) interfaced with a computer, at ×40 magnification. Collagen thickness was measured using cellSens (Olympus, Tokyo, Japan). Three fields from each section were selected, and the collagen thickness was measured.

### 2.8. Elastic Fiber Staining

To visualize elastic fibers, sections were stained with resorcin-fuchsin for 60 min prior to staining with hematoxylin and eosin. The specimens were examined and photographed using a digital microscope camera (DP21, Olympus, Tokyo, Japan) interfaced with a computer at ×200 magnification.

### 2.9. Immunohistochemical Staining for Hyaluronic Acid

Sections were stained using a biotinylated hyaluronic acid binding protein probe [14]. Briefly, tissue sections were treated with protease I (Roche, Basel, Switzerland) at 37 °C for 5 min for antigen retrieval. Biotinylated hyaluronic acid binding protein (1:50; Hokudo, Hokkaido, Japan) in PBS was then added, and the slides were kept in a humid chamber at 4 °C overnight. The tissue sections were then incubated for 60 min with peroxidase-conjugated streptavidin (GTX85912; Gene Tex, Irvine, CA, USA 1:200 dilution). The sites of peroxidase activity were determined using diaminobenzidine (DAKO EnVision™ detection system, Agilent, Santa Clara, CA, USA) as the substrate. Tissue sections that were not treated with biotinylated hyaluronic acid binding protein were used as negative controls. The specimens were examined and photographed using a digital microscope camera (DP21, Olympus, Tokyo, Japan) interfaced with a computer at ×100 magnification.

### 2.10. Statistical Analysis

Results are expressed as means ± standard errors of the mean of at least three independent experiments. Statistical analyses were performed using BellCurve for Excel ver. 2.13 software (Social Survey Research Information Co., Ltd., Tokyo, Japan) by Kruskal–Wallis analysis with the Steel post-hoc test. *p* < 0.05 was considered to indicate statistical significance.

## 3. Results

### 3.1. Microarray Profiling of TIG113 Cells Exposed to BCE

Our previous studies showed that 1.0 μg/mL of BCE exerts a phytoestrogenic effect [23,24]. Therefore, we initially compared gene expression in TIG113 cells before and after exposure to 1.0 μg/mL BCE using microarrays. IPA was performed to investigate the functional relationships between sets of genes showing altered expression levels. Several predicted upstream regulators were detected, but only E2 had a z-score >2.0 (z-score = 2.68) (Table 1). In addition, the expression of genes downstream to estradiol was markedly affected by BCE treatment (Table S1). The z-score of estrogen receptor (ER)α was 1.68, whereas that of ERβ was 0.89. These results show that BCE acted similarly to estradiol in TIG113 cells. Detailed expression changes were assessed for the gene sets regulated by estradiol and ERα (Tables S1 and S2). These results indicate that BCE significantly altered the expression levels of genes involved in pathways related to estrogen signaling. The whole transcript microarray analysis of TIG113 cells exposed to BCE (1.0 μg/mL) showed that *COL3A1*, *HAS3*, and *TIMP3* were upregulated >1.5-fold, although the expression of other ECM genes was not altered (Table 2). The levels of *HYAL3* and *MMP12*, which are involved in ECM protein breakdown, decreased in the presence of BCE.

Ingenuity® Pathway Analysis (IPA) software can analyze biological pathways based on microarray data. Genes showing ≥1.5-fold up or downregulation following exposure of TIG113 cells to 1.0 μg/mL BCE were analyzed using IPA software. The z-score algorithm was utilized to reduce the possibility of false-positive results, where z ≥ 2.0 indicated a significant increase in transcript expression.

### 3.2. RT-qPCR Analysis of ECM Gene mRNA Levels

**Figure 1:**
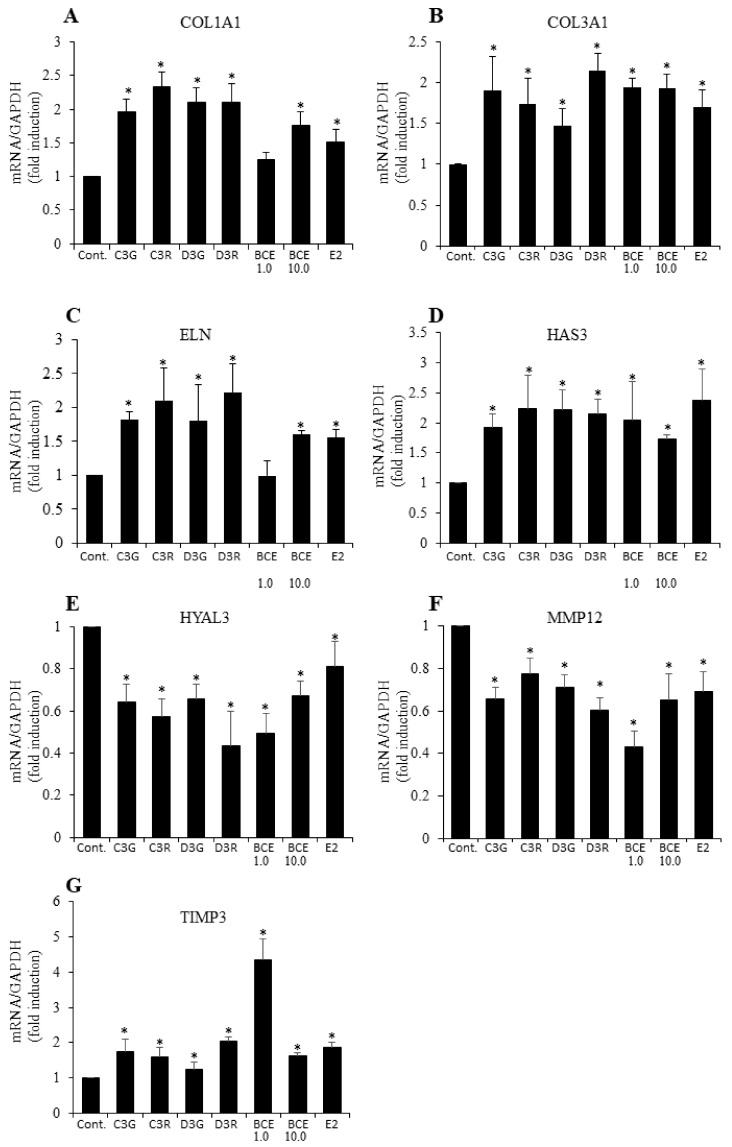
Effects of blackcurrant anthocyanins and 17β-estradiol (E2) on mRNA levels of estrogen-dependent genes. TIG113 cells were treated with the indicated anthocyanins (10 μM), BCE (1.0 or 10.0 μg/mL), or E2 (1 nM) for 24 h. mRNA levels of (**A**) *COL1A1*, (**B**) *COL3A1*, (**C**) *ELN*, (**D**) *HAS3*, (**E**) *HYAL3*, (**F**) *MMP12*, and (**G**) *TIMP3* were quantified by RT-qPCR. Data represent the means ± standard errors of the mean of at least three independent experiments; * *p* < 0.05 vs. untreated control cells.

To confirm the anthocyanin- or BCE-mediated effects on the expression of genes determined to be upregulated by ≥1.5 fold by microarray (Table 2) and synthesis or degradation genes related to ECM components, TIG113 cells were incubated with 10 μM anthocyanins, 1.0 or 10.0 μg/mL of BCE (these concentrations of compounds had a phytoestrogen effect in our previous studies [23,24]), or 1 nM of E2 for 24 h. The RT-qPCR analysis showed that these anthocyanin and BCE concentrations upregulated *COL1A1*, *COL3A1*, *ELN, HAS3*, and *TIMP3* mRNA. *COL1A1* and *ELN* mRNA levels were not increased under the microarray condition (1 μg/mL); however, they were increased at 10 μg/mL. In contrast, the *HYAL3* and *MMP12* mRNA levels were significantly decreased under these conditions (Figure 1). These results suggest that ECM protein expression was induced following exposure to BCE due to activation of the estradiol pathway.

### 3.3. Induction of ECM Proteins by BCE and Anthocyanins

**Figure 2:**
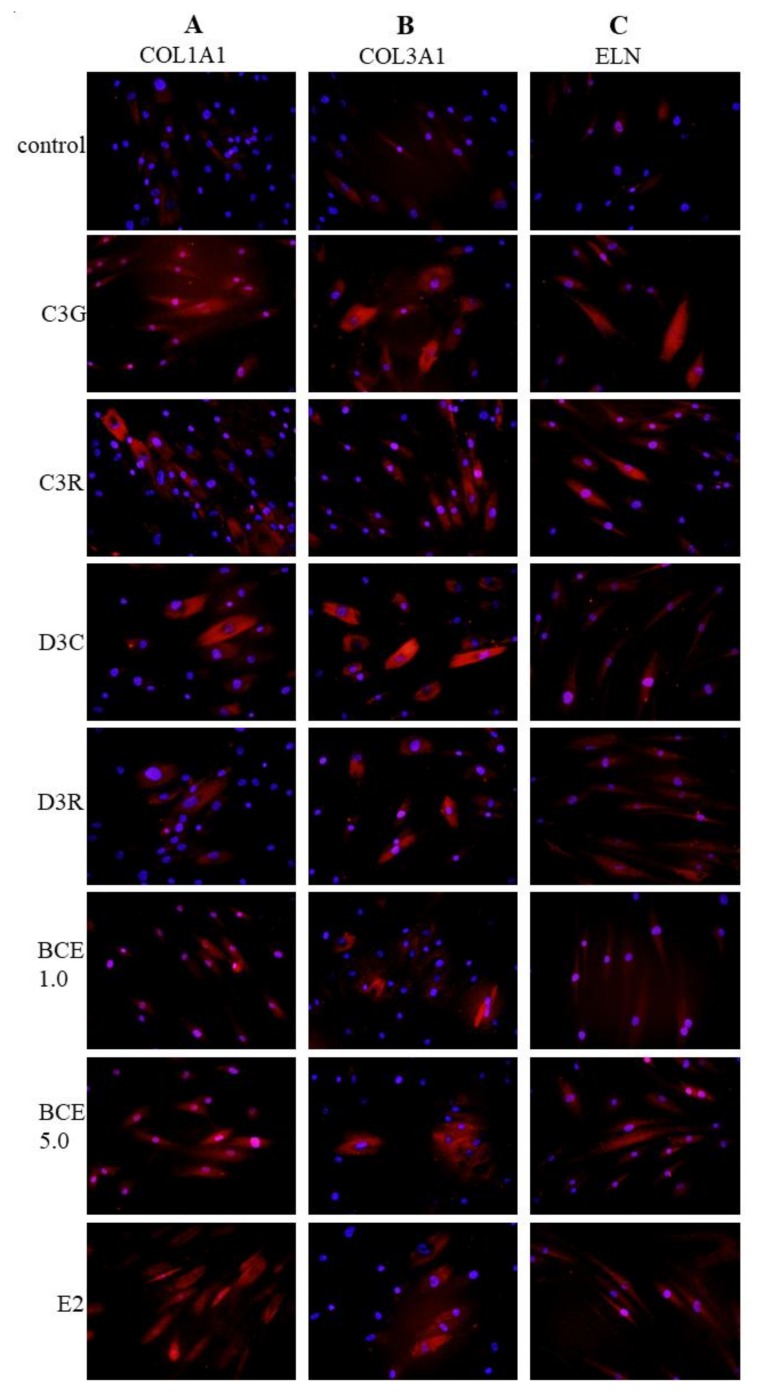
Effects of blackcurrant anthocyanins and E2 on the protein levels of COL1A1 (**A**), COL3A1 (**B**), and elastin (**C**). TIG113 cells were treated with the indicated anthocyanins (10 μM), BCE (1.0 or 5.0 μg/mL), or E2 (1 nM) for 48 h. Cells were stained, as indicated, using an anti-COL1A1, anti-COL3A1, or anti-elastin antibody (red signals). The nuclei were visualized using DAPI (4′,6-diamidino-2-phenylindole, blue). Images are at 200× magnification.

Immunofluorescence staining was performed to investigate whether the level of ECM proteins was increased by treatment with BCE or anthocyanins. These analyses revealed that the protein levels of COL1A1 (Figure 2A), COL3A1 (Figure 2B), and elastin (Figure 2C) were increased in the cytoplasm of TIG113 exposed to the anthocyanins or BCE, compared to the levels in untreated control cells (Figure 2).

### 3.4. Increased Collagen Thickness in BCE-Treated OVX Rats

**Figure 3:**
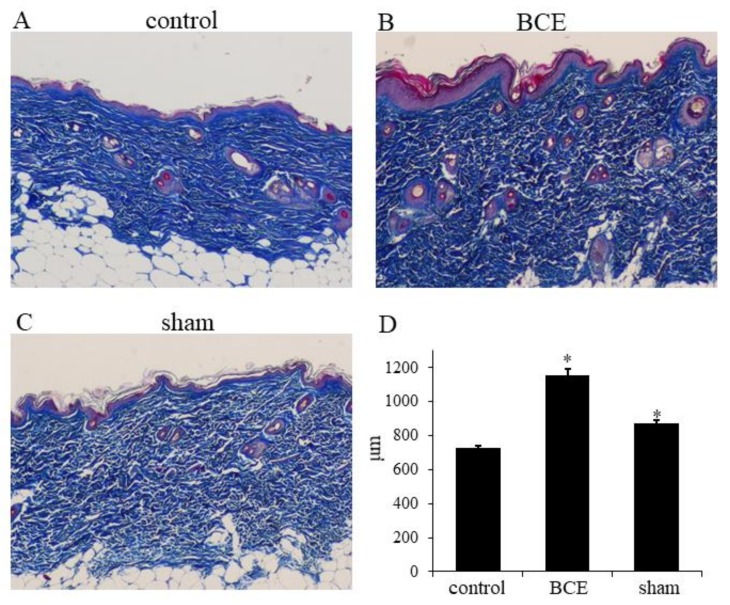
Light microscopy of Masson’s trichrome staining of skin in control (OVX + 0% BCE; (**A**)), OVX + 3% BCE (**B**), and sham surgery rats (**C**). Images are at original magnification: ×40. (**D**) Quantification of the thickness (μm) of the collagen layer; * *p* < 0.05 vs. control (untreated OVX rats).

Because the levels of ECM mRNAs and proteins were increased in TIG113 cells exposed to anthocyanins or BCE, we also investigated the in vivo effects on OVX rat skin, using Masson’s trichrome stain. Collagen thickness was significantly greater in OVX rats treated with 3% BCE (1156 ± 36 μm) and in sham surgery rats (845 ± 15 μm) than in untreated OVX rats (control) (726 ± 16 μm) (*P* < 0.01) (Figure 3A–D).

### 3.5. Induction of Elastic Fibers in BCE-Treated OVX Rats

**Figure 4:**
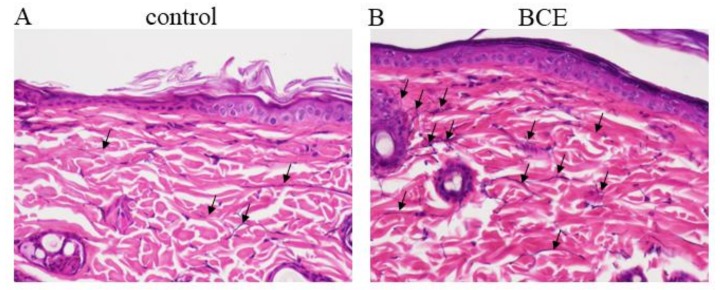

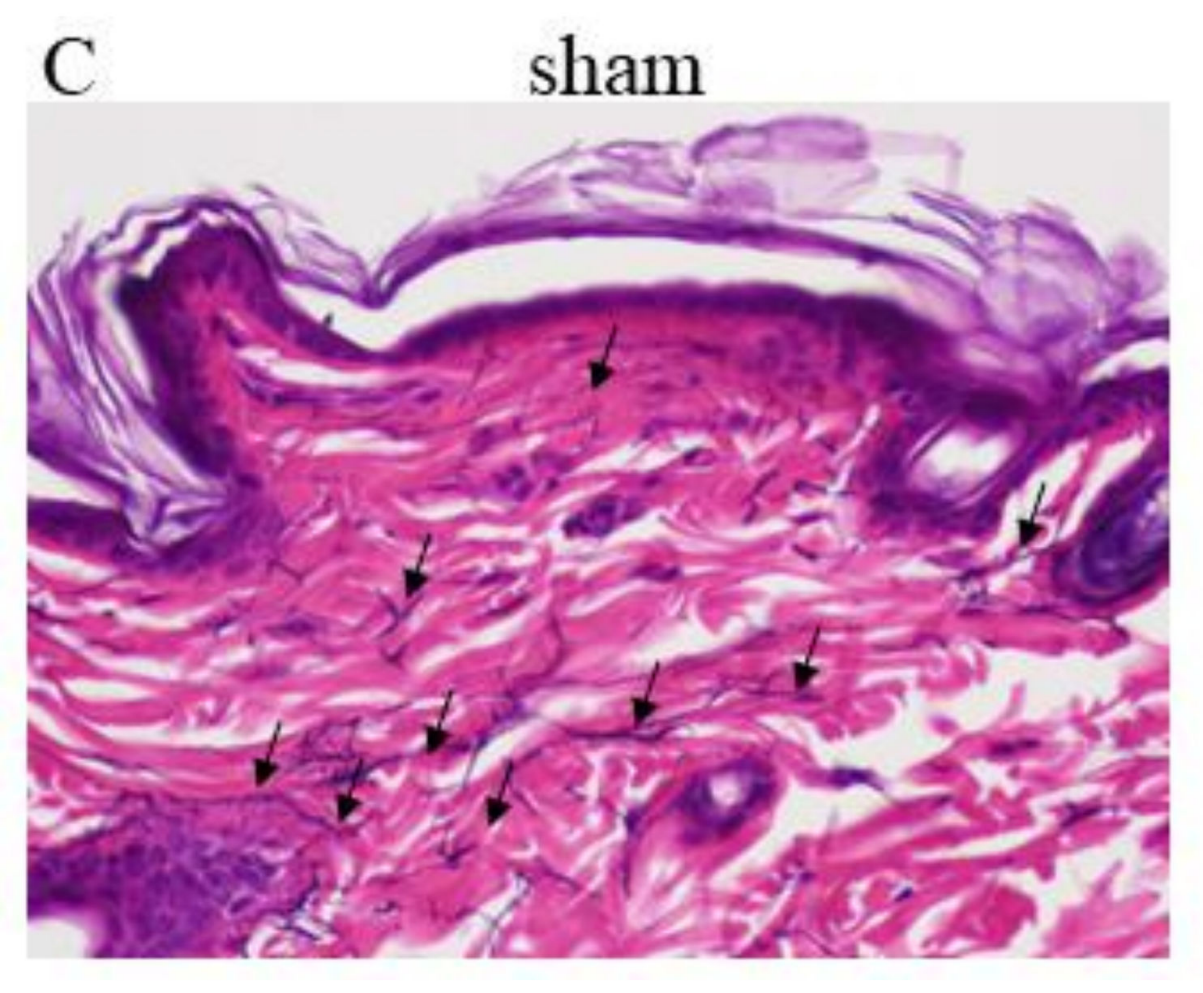
Light microscopy image of elastic fiber staining of skin from control (OVX + 0% BCE; (**A**)), OVX + 3% BCE (**B**), and sham surgery rats (**C**). Images are at 200× magnification.

Elastic fibers were identified in the skin by modified Elastica van Gieson staining. The elastic fiber content was clearly greater in OVX rats treated with 3% BCE and in sham surgery rats than in untreated OVX rats (control) (Figure 4A–C, arrows).

### 3.6. Induction of Hyaluronic Acid Expression in BCE-Treated OVX Rats

**Figure 5:**
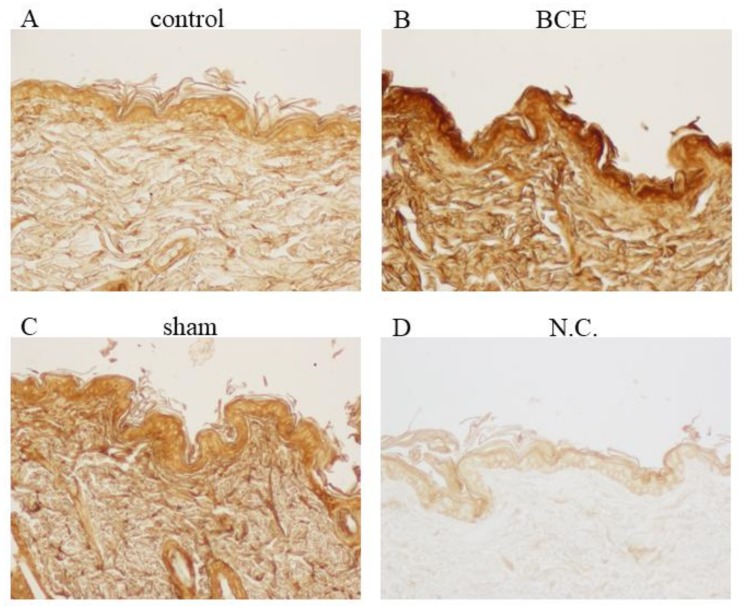
Light microscopy images of biotinylated hyaluronic acid-binding protein staining of skin from control (OVX + 0% BCE; (**A**)), OVX + 3% BCE (**B**), and sham surgery rats (**C**). Tissue sections from sham surgery rats that were not treated with binding protein were used as the negative controls (N.C.; (**D**)). Images are at 100× magnification.

The immunohistochemical analysis of rat skin tissue indicated that treatment of OVX rats with 3% BCE increased the level of hyaluronic acid in the ECM (Figure 5A–D). Furthermore, the level of hyaluronic acid was higher in BCE-treated OVX rats than in the sham surgery rats.

## 4. Discussion

This study examined the effects of blackcurrant anthocyanin phytoestrogens in the skin. Exposure of the human skin fibroblast TIG113 cell line to BCE altered the mRNA expression of genes activated by estradiol and ERα signaling (Tables S1 and S2). Skin fibroblasts have been reported to express ERs, and ERα and ERβ contribute to the production of the ECM within the skin. There are reports indicating that ERβ is expressed more frequently by skin fibroblasts than ERα [30], although one study did not observe ERβ expression [31]. Expression of ERα or β by fibroblasts is known to vary depending on the type and age of the skin. The TIG113 cells used in this study were derived from the female precordium and did not express ERβ, whereas ERα mRNA was expressed robustly (data not shown). This suggests that the observed upregulation of genes downstream of estradiol by BCE was due to ERα activation. The IPA also yielded a greater z-score for ERα than for ERβ (Table 1), providing further evidence that BCE-associated phytoestrogenic activity was mediated by ERα signaling in TIG113 cells.

The mRNA levels of molecules related to the ECM (such as *COL1A1*, *COL3A1*, *ELN,* and *HAS3*) increased following exposure to BCE, whereas that of the degrading enzyme, *MMP12*, decreased. The expression of *TIMP3* was increased by BCE treatment in TIG113 cells. TIMP is an MMP inhibitor, and estrogen is known to be involved in TIMP–MMP balance and collagen degradation in the OVX rat [32]. In addition, ECM protein levels increased consistently. MMP2, 9, and 12 are known to degrade elastin [33,34]. A decreased level of MMP12 in TIG113 cells would, therefore, reduce the degradation of elastin, whereas increased *ELN* mRNA levels would be predicted to increase elastin production. No changes were observed in the expression of other MMPs, but an increased TIMP3 level would likely suppress MMP activity, suggesting that the increase in ECM production was due to synergistic effects on these genes. The expression of some genes, such as *TIMP3* and *MMP12*, did not change BCE in a concentration dependent manner. This suggests that the optimal concentration of BCE differs depending on the gene. In vitro, estrogen treatment was reported to promote ECM synthesis or reduce MMP production in fibroblasts [12,25,26], although other studies have not observed these effects [31]. This may reflect the different anatomical origins of the fibroblasts employed in the studies, as it may influence the ER expression level and the responsiveness to estrogen. Transforming growth factor-β (TGF-β) is important for biosynthesis of the ECM [35,36,37]. Estrogen is known to crosstalk with the TGF-β signaling pathway [38]. Our previous study also suggested that BCE induces the expression of genes downstream of TGF-β in breast cancer cells expressing high levels of ERα (MCF7), and that a similar phenomenon occurs in TIG113 cells [24]. In the present study, IPA revealed an altered expression of genes downstream of TGF-β (Supporting Information Table S3), and the level of TGF-β3 mRNA was also increased (Tables S1 and S2).

In this study, the levels of insulin-like growth factor (IGF)-2, IGF binding protein (IGFBP)2, and IGFBP5 were greatly increased in TIG113 cells treated with BCE (Table S1). IGF has a structure very similar to that of insulin, and it is produced in tissues such as the liver and skeletal muscle following exposure to growth hormone. Furthermore, human dermal fibroblasts produce IGF-1 and IGF-2 when treated with various factors, including growth hormone. IGF-1 and IGF-2 have an approximately 60% amino acid sequence homology with insulin [39]. IGF-1 has been shown to reduce wrinkles in the skin and therefore, is an effective cosmetic treatment agent [40,41]. In human skin fibroblasts, the expression of IGFBP is regulated by factors, such as IGFs, TGF-β1, estradiol, and testosterone. IGF and IGFBPs promote dermal fibroblast cell proliferation, migration, and production of growth factors such as TGF-β1 [42,43,44]. Because IGF-2 and IGFBP2 are estrogen-responsivefactors [45], these findings suggest that phytoestrogens within BCE can activate estrogen signaling, as well as increase the levels of IGF, IGFBP, and TGF-β1. In addition, estradiol and epidermal growth factor/IGF signaling influence the synthesis of proteoglycans [46]. The beneficial effects of BCE on the skin might reflect its synergistic effects on the expression of these factors.

In vivo, we investigated whether BCE acts as a phytoestrogen by examining changes in the ECM in OVX rats. These rats do not secrete estrogen and therefore, are considered a model of menopause. After a 3-month treatment period, skin collagen, elastin, and hyaluronic acid levels decreased in the untreated OVX rats. However, the addition of 3% BCE to the OVX rat diet maintained the levels of these ECM proteins in the dermis. These results are consistent with reports that collagen and hyaluronic acid levels increased when OVX rats and mice were treated with estrogen and genistein [27,47]. In vivo, levels of these ECM proteins tended to be higher in OVX rats treated with 3% BCE than in the sham surgery rats (Figure 3, Figure 4 and Figure 5). Furthermore, our in vitro findings showed that in some situations, the effects on ECM gene expression were greater in BCE- and anthocyanin-treated cells than in cells exposed to E2 (Figure 1 and Figure 2). Gopaul et al. also reported that the phytoestrogen, equol, promoted the induction of ECM protein expression to a greater extent than estrogen [25]. In addition to interacting with the ERα, BCE may act on other receptors. For example, estrogen-related receptor alpha is an orphan receptor; estrogen is not a ligand for this receptor, but phytoestrogens such as genistein have been shown to act as its ligands [48]. Therefore, BCE may exert greater effects than estrogen by inducing complex activation of, and cross-talk between, downstream genes and receptor signaling pathways. Previous studies have shown that the anthocyanin dose that was used in this study exerts a phytoestrogenic effect [23,24,27]. We believe that it is difficult to ingest the amount of anthocyanin used in this study by consuming blackcurrant fruit through the diet. However, the consumption of this amount seems possible with anthocyanin-enriched blackcurrant supplements.

Estrogen was reported to exert a greater effect on ECM production in fibroblasts cocultured with keratinocytes than in fibroblasts cultured alone [47]. The in vivo experimental results may therefore be influenced by the cytokines secreted by keratinocytes [49].

In addition, osteoporosis can develop in OVX rats and menopausal women. Phytoestrogens and BCE were reported to attenuate osteoporosis in OVX rats [50], indicating that BCE may have phytoestrogen activity in bones as well as in the skin.

## 5. Conclusions

The present study demonstrates the phytoestrogenic activity of BCE and anthocyanins in the skin. Exposure to BCE or anthocyanins increased the level of ECM molecules such as collagen and elastin in human skin fibroblasts. In addition, BCE increased the ECM component levels in OVX rat skin. To the best of our knowledge, this is the first report of the phytoestrogen activity of BCE and anthocyanins in the skin. In this study, we did not administer blackcurrants to humans, but long-term ingestion is expected to be effective, even at low concentrations. We will study this in future research.

## Figures and Tables

**Table 1:** IPA of blackcurrant extract (BCE)-treated normal human female skin fibroblast cell line (TIG113) cells.

Predicted Upstream Regulator	z-Score
Estradiol	2.68
ERα	1.68
ERβ	0.89

**Table 2:** Expression of extracellular matrix (ECM) genes in BCE-treated TIG113 cells.

Abbreviation	Full Name	Fold Change *
*COL1A1*	collagen, type I, alpha 1	1.0
*COL1A2*	collagen, type I, alpha 2	1.0
*COL3A1*	collagen, type III, alpha 1	1.8
*ELN*	elastin	−1.4
*HAS2*	hyaluronan synthase 2	1.3
*HAS3*	hyaluronan synthase 3	1.5
*HYAL2*	hyaluronoglucosaminidase 2	−1.3
*HYAL3*	hyaluronoglucosaminidase 3	−1.6
*HYAL4*	hyaluronoglucosaminidase 4	−1.1
*MMP1*	matrix metalloproteinase 1	−1.4
*MMP2*	matrix metalloproteinase 2	−1.0
*MMP3*	matrix metalloproteinase 3	1.3
*MMP9*	matrix metalloproteinase 9	−1.1
*MMP12*	matrix metalloproteinase 12	−1.5
*TIMP1*	tissue inhibitor of metalloproteinase 1	−1.1
*TIMP2*	tissue inhibitor of metalloproteinase 2	1.1
*TIMP3*	tissue inhibitor of metalloproteinase 3	5.7
*TIMP4*	tissue inhibitor of metalloproteinase 4	1.2

* Alterations of mRNA expression in TIG113 cells treated with BCE (1.0 μg/mL for 24 h) by microarray analysis.

## Supplementary Materials

Supplementary material can be found at http://www.mdpi.com/2072-6643/10/4/495/s1. Table S1: Effects of BCE on estradiol-regulated gene expression in TIG113 cells, Table S2: Effects of BCE on ESR1-regulated gene expression in TIG113 cells, Table S3: Effects of BCE on TGFβ1-regulated gene expression in TIG113 cells.

## Abbreviations

BCE	blackcurrant extract
E2	17β-estradiol
C3G	cyanidin-3-glucoside
C3R	cyanidin-3-rutinoside
D3G	delphinidin-3-glucoside
D3R	delphinidin-3-rutinoside
ER	estrogen receptor
ECM	extracellular matrix
GAPDH	glyceraldehyde 3-phosphate dehydrogenase
HAS	hyaluronan synthase
HYAL	hyaluronoglucosaminidase
IGF	insulin-like growth factor
IGFBP	insulin-like growth factor binding protein
IPA	Ingenuity^®^ Pathway Analysis
MMP	matrix metalloproteinase
TGF	transforming growth factor
TIMP	tissue inhibitor of metalloproteinase
OVX	ovariectomized
