# Supplementary material for: Blackcurrant Anthocyanins Increase the Levels of Collagen, Elastin, and Hyaluronic Acid in Human Skin Fibroblasts and Ovariectomized Rats
Source: Nutrients. 2018 Apr 16;10(4):495. doi: 10.3390/nu10040495 (PMC5946280; doi:10.3390/nu10040495)
Supplement: Supplementary file 1 [file nutrients-10-00495-s001.pdf]

nutrients

MDPI

Supplementary Materials

Table S1. Effects of BCE on estradiol-regulated gene expression in TIG113 cells.

Gene Symbol	Gene Name	Fold Change
IGFBP2	insulin like growth factor binding protein 2	115.3
IGF2	insulin like growth factor 2	26.6
WNT11	Wnt family member 11	16.9
AQP1	aquaporin 1	16.7
TTR	transthyretin	14.2
EGR2	early growth response 2	12.3
CLCA2	chloride channel accessory 2	10.5
WNT5A	Wnt family member 5A	8.4
PLXDC1	plexin domain containing 1	7.7
CRABP2	cellular retinoic acid binding protein 2	7.6
LTBP1	latent transforming growth factor beta binding protein 1	7.5
TH	tyrosine hydroxylase	6.4
SFRP1	secreted frizzled related protein 1	6.2
PDK4	pyruvate dehydrogenase kinase 4	5.9
TACSTD2	tumor associated calcium signal transducer 2	5.7
TIMP3	tissue inhibitor of metalloproteinase 3	5.7
EGR1	early growth response 1	5.2
INHBA	inhibin beta A subunit	5.0
COLEC12	collectin subfamily member 12	4.7
ST8SIA1	ST8 alpha-N-acetyl-neuraminide	4.4
TRIB2	tribbles pseudokinase 2	3.7
TMEM2	transmembrane protein 2	3.4
PLAU	plasminogen activator, urokinase	3.3
SPRY1	sprouty RTK signaling antagonist 1	3.1
TIAM1	T-cell lymphoma invasion and metastasis 1	3.1
LOXL4	lysyl oxidase like 4	3.0
TFAP2C	transcription factor AP-2 gamma	2.8
NR2F1	nuclear receptor subfamily 2 group F member 1	2.7
NEDD9	neural precursor cell expressed, developmentally down-regulated 9	2.7
PTGER3	prostaglandin E receptor 3	2.6
TP53I11	tumor protein p53 inducible protein 11	2.6
CFB	complement factor B	2.6
FBLN1	fibulin 1	2.6
PENK	proenkephalin	2.5
HLA-DRB1	major histocompatibility complex, class II, DR beta 1	2.4
VEGFA	vascular endothelial growth factor A	2.4
CTGF	connective tissue growth factor	2.3
THBS4	thrombospondin 4	2.2
KLF10	kruppel like factor 10	2.2
IGFBP5	insulin like growth factor binding protein 5	2.1
MAOA	monoamine oxidase A	2.1
TGFB3	transforming growth factor beta 3	2.1
ITGA1	integrin subunit alpha 1	2.1
LOC102724428	serine/threonine-protein kinase SIK1	2.1
PITX1	paired like homeodomain 1	2.0
VDR	vitamin D receptor	2.0
KLF6	kruppel like factor 6	-2.2

Table S2. Effects of BCE on ESR1-regulated gene expression in TIG113 cells.

Gene Symbol	Gene Name	Fold Change
IGFBP2	insulin like growth factor binding protein 2	115.3
WNT11	Wnt family member 11	16.9
ROBO2	roundabout guidance receptor 2	8.0
PLXDC1	plexin domain containing 1	7.7
CRABP2	cellular retinoic acid binding protein 2	7.6
LTBP1	latent transforming growth factor beta binding protein 1	7.5
MAP2	microtubule associated protein 2	6.2
PDK4	pyruvate dehydrogenase kinase 4	5.9
EPHX4	epoxide hydrolase 4	5.7
ANK3	ankyrin 3	5.6
EGR1	early growth response 1	5.2
FGFR2	fibroblast growth factor receptor 2	4.8
ICOSLG/LOC102723996	inducible T-cell costimulator ligand	4.6
VAV3	vav guanine nucleotide exchange factor 3	4.2
ODF3B	outer dense fiber of sperm tails 3B	3.9
CCL2	C-C motif chemokine ligand 2	3.5
CHST2	carbohydrate sulfotransferase 2	3.4
TMEM2	transmembrane protein 2	3.4
PLAU	plasminogen activator, urokinase	3.3
CEBPA	CCAAT/enhancer binding protein alpha	3.2
ASS1	argininosuccinate synthase 1	3.2
CPE	carboxypeptidase E	3.1
LOXL4	lysyl oxidase like 4	3.0
TGM2	transglutaminase 2	3.0
PDGFD	platelet-derived growth factor D	2.9
TFAP2C	transcription factor AP-2 gamma	2.8
NEDD9	neural precursor cell expressed, developmentally downregulated 9	2.7
PTGER3	prostaglandin E receptor 3	2.6
TP53I11	tumor protein p53 inducible protein 11	2.6
FBLN1	fibulin 1	2.6
PRKCH	protein kinase C eta	2.5
MME	membrane metalloendopeptidase	2.4
VEGFA	vascular endothelial growth factor A	2.4
SLC39A8	solute carrier family 39 member 8	2.4
THRB	thyroid hormone receptor beta	2.3
DOCK4	dedicator of cytokinesis 4	2.3
THBS4	thrombospondin 4	2.2
IER3	immediate early response 3	2.2
SERPINB9	serpin family B member 9	2.2
IGFBP5	insulin like growth factor binding protein 5	2.1
CSAD	cysteine sulfinic acid decarboxylase	2.1
TGFB3	transforming growth factor beta 3	2.1
TMSB15A	thymosin beta 15a	2.1
PLCE1	phospholipase C epsilon 1	-2.0
PRLR	prolactin receptor	-2.0
S100P	S100 calcium binding protein P	-2.0
SLC7A5	solute carrier family 7 member 5	-2.1
GSG1	germ cell associated 1	-2.1
TM4SF1	transmembrane 4 L six family member 1	-2.3
KRT19	keratin 19	-2.4

Table S3. Effects of BCE on TGFβ1-regulated gene expression in TIG113 cells.

Gene Symbol	Gene Name	Fold Change
WNT11	Wnt family member 11	16.9
ASPN	asporin	15.7
RASGRP3	RAS guanyl-releasing protein 3	9.5
WNT5A	Wnt family member 5A	8.4
LTBP1	latent transforming growth factor beta binding protein 1	7.5
TIMP3	TIMP metallopeptidase inhibitor 3	5.7
RGCC	regulator of cell cycle	5.2
EGR1	early growth response 1	5.2
INHBA	inhibin beta A subunit	5.0
OLFM2	olfactomedin 2	4.7
ANGPTL4	angiopoietin like 4	3.8
NOX4	NADPH oxidase 4	3.7
CCL2	C-C motif chemokine ligand 2	3.5
PLAU	plasminogen activator, urokinase	3.3
CEBPA	CCAAT/enhancer binding protein alpha	3.2
PIM1	Pim-1 proto-oncogene, serine/threonine kinase	3.2
ASS1	argininosuccinate synthase 1	3.2
PRDM1	PR/SET domain 1	3.1
TGM2	transglutaminase 2	3.0
TNC	tenascin C	2.8
NEDD9	neural precursor cell expressed, developmentally downregulated 9	2.7
COL7A1	collagen type VII alpha 1 chain	2.6
CD14	CD14 molecule	2.5
CAMK2N1	calcium/calmodulin dependent protein kinase II inhibitor 1	2.4
FERMT1	fermitin family member 1	2.4
VEGFA	vascular endothelial growth factor A	2.4
PMEPA1	prostate transmembrane protein, androgen induced 1	2.3
DOCK4	dedicator of cytokinesis 4	2.3
CTGF	connective tissue growth factor	2.3
IER3	immediate early response 3	2.2
KLF10	kruppel like factor 10	2.2
FABP5	fatty acid binding protein 5	2.1
ITGA1	integrin subunit alpha 1	2.1
INPP5D	inositol polyphosphate-5-phosphatase D	2.1
PIK3CD	phosphatidylinositol-4,5-bisphosphate 3-kinase catalytic subunit delta	2.0
VDR	vitamin D receptor	2.0
